# Biopsychosocial intervention for stroke carers (BISC): results of a feasibility randomised controlled trial and nested qualitative interview study

**DOI:** 10.1177/0269215520937039

**Published:** 2020-06-21

**Authors:** Marion F Walker, Sheila Birchall, Christine Cobley, Laura Condon, Rebecca Fisher, Joanna Fletcher-Smith, Miriam R Golding-Day, Christopher Greensmith, Eirini Kontou, Oliver Matias, Nikola Sprigg, Shirley A Thomas, Phillip J Whitehead

**Affiliations:** 1 Division of Rehabilitation, Ageing and Wellbeing, University of Nottingham, Nottingham, UK; 2 Department of Clinical Psychology, Derbyshire Healthcare NHS Foundation Trust, Derby, UK; 3 IAPT Service, Nottinghamshire Healthcare NHS Foundation Trust, UK; 4 Division of Clinical Neuroscience, University of Nottingham, Nottingham, UK; 5 Department of Social Work, Education and Community Wellbeing, Northumbria University, Newcastle-Upon-Tyne, UK

**Keywords:** Stroke, carers, biopsychosocial, complex intervention, randomised controlled trial

## Abstract

**Objective:**

To determine the feasibility of recruiting to and delivering a biopsychosocial intervention for carers of stroke survivors.

**Design:**

Feasibility randomised controlled study with nested qualitative interview study.

**Setting:**

The intervention was delivered in the community in either a group or one-to-one format.

**Subjects:**

Carers and stroke survivors within one year of stroke onset.

**Interventions:**

A carer targeted intervention delivered by a research psychologist in six structured two-hour sessions or usual care control. The intervention combined education about the biological, psychological and social effects of stroke with strategies and techniques focussing on adjustment to stroke and caregiving. Stroke survivors in both groups received baseline and follow-up assessment but no intervention.

**Main Outcome:**

Recruitment rate, study attrition, fidelity of intervention delivery, acceptability and sensitivity of outcome measures used (health related quality of life, anxiety and depression and carer burden six months after randomisation).

**Results:**

Of the 257 carers approached, 41 consented. Six withdrew before randomisation. Eighteen participants were randomised to receive the intervention and 17 to usual care. Attendance at sessions was greater when treated one-to-one. Feedback interviews suggested that participants found the intervention acceptable and peer support particularly helpful in normalising their feelings. Thirty participants were assessed at follow-up with improvements from baseline on all health measures for both groups.

**Conclusions:**

Our results suggest that a biopsychosocial intervention was acceptable to carers and can be delivered in group and one-to-one formats. Timing of approach and mode of intervention delivery is critical and requires tailoring to the carers individual needs.

## Introduction

Almost a third of stroke survivors who leave hospital needing help with activities of daily living, receive this help from informal carers.^1^ The sudden onset of stroke means that friends and family of stroke survivors can often find themselves in new and unexpected situations undertaking roles for which they have received little preparation, training or warning.^2^ Carers of stroke survivors may therefore have specific and unique needs and requirements of their own. Although being a carer for a stroke survivor can be a positive and rewarding experience,^3^ it may also be daunting and challenging. Studies of stroke carers report that many carers experience: anxiety, frustration, sleep disturbance, stress and depression.^4,5^ National stroke guidelines have emphasised the importance of providing support and intervention for carers,^6,7^ however health care attention has been primarily directed towards the stroke survivor’s recovery and rehabilitation leaving limited time to address the carer’s needs.

In a large multicentre randomised controlled trial evaluating a structured training programme to carers of inpatient stroke survivors,^8^ the authors found that compliance with the intervention delivered varied greatly and that there were no differences in outcomes for carers or stroke survivors. The authors also concluded that the initial post-stroke inpatient period might not be the optimum timepoint to deliver the intervention.

High quality evidence to support family caregivers has been lacking,^9^ with authors calling for more rigorous study designs paying particular attention to defining the content of the intervention, the fidelity of the intervention delivery and the sustainability of the outcomes.^10^

A systematic review of non-pharmacological interventions for carers of stroke survivors found insufficient evidence to support the use of ‘information and support’ or ‘psychoeducational’ interventions for stroke carers.^11^ A subsequent review of psychosocial interventions for stroke family carers concluded that more randomised controlled trials of such programmes were needed.^12^

A more recent systematic review and meta-analysis of stroke survivors, carers and survivor-carer dyads incorporating thirty-one randomised controlled trials^13^ found in six trials that psychosocial interventions reduced depressive symptoms in carers. Authors also noted that more research was required to investigate interventions that improved quality of life and coping mechanisms for carers. Whilst these reviews specifically addressed psychoeducational and psychosocial interventions none specifically addressed the importance of the interaction with physical health.

We developed an intervention designed to equip carers of stroke survivors to address their own health needs, with additional strategies and techniques which focussed on the development of coping mechanisms and adjustment to their new role with the potential to impact on future quality of life. The intervention combined education about the biological, psychological and social effects of caring for stroke with strategies and techniques focussing on successful adjustment to stroke and caregiving. The intervention is grounded in the biopsychosocial model of health and illness which posits that psychobiological vulnerability is influenced by an interaction of biological (physical health), psychological (thoughts, emotions and behaviours) and social (relationships and roles) factors.^14^ The intervention development and description of content adhered to the international consensus-based recommendations on the development, monitoring and reporting of stroke rehabilitation research^15^ and is described in detail elsewhere.^16^

The primary objective of the Biopsychosocial Intervention for Stroke Carers (BISC) study was to evaluate whether it was feasible to deliver this novel intervention to carers of stroke survivors as part of a randomised controlled trial in a UK setting. The protocol was published prospectively.^17^ Specific objectives for this feasibility randomised controlled trial were:

1. To ascertain whether stroke carers and stroke survivors were willing to be recruited and randomised.
2. To identify consent and attrition rates.
3. To determine whether the intervention could be delivered as planned.
4. To evaluate whether the intervention was acceptable to participants.
5. To determine the appropriateness, suitability and sensitivity of outcome measures for use in a larger study.

## Method

**Figure 1.**
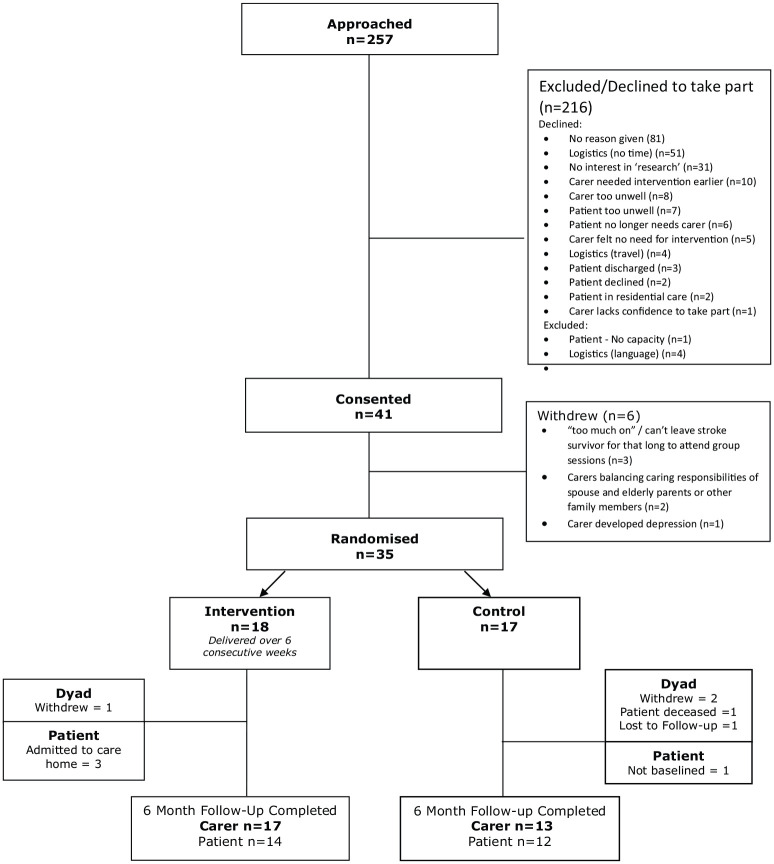
Flow diagram showing participant’s progress using the CONSORT template.^18^

We conducted a single centre feasibility randomised controlled trial with nested qualitative interview study (Figure 1). The study was funded by the National Institute for Health Research (Research for Patient Benefit Programme, Biopsychosocial Intervention for Stroke Carers (BISC), PB-PG-0613-31064) and opened for recruitment on 1st November 2015 closing on 28th June 2017. Favourable ethical opinion was provided by the East Midlands – Nottingham 2 Research Ethics Committee (14/EMI/1264). The trial is registered with the ISRCTN registry (ISRCTN15643456) and sponsored by the University of Nottingham. This paper has been written in accordance with the CONSORT 2010 statement: extension to randomised pilot and feasibility trials.

The randomised controlled trial was a parallel group, two arm trial with a 1:1 allocation ratio to biopsychosocial intervention: usual care control. The setting for recruitment was a stroke unit at a large teaching hospital, community stroke services, stroke clubs and support groups. The setting for intervention delivery was a social centre used by the local community. Our aim was to recruit up to 40 dyads (20 in each arm of the trial) to test the randomisation process and the feasibility of delivering the intervention. Informed by findings from earlier research,^8^ participants included carers of individuals who had sustained a stroke within the past year. Our definition of a carer was a family member or friend who was/would be providing support for a stroke survivor who would have otherwise experienced considerable difficulty managing without the assistance and support of their carer due to their stroke condition. Stroke survivors were recruited along with their carer, however, only the carer received the intervention.

The inclusion criteria were:

*Stroke carers*:

- Aged 18 or over
- Carer of a person with a confirmed diagnosis of stroke within one year of stroke onset
- Capacity to provide informed consent
- Willing to attend a six-week intervention programme

*Stroke survivors*:

- Aged 18 or over
- Confirmed diagnosis of stroke made by a stroke physician
- Within one year of stroke onset
- Capacity to provide informed consent or consultee opinion that the person would wish to participate

The exclusion criteria were:

*Stroke carers*:

- Unable to speak English
- Engaged in other research involving biopsychosocial/psychological interventions
- People with visual (blindness) or auditory (deafness) impairments that would preclude them from participating in the intervention sessions.

*Stroke survivors*:

- Unable to speak English
- People engaged in other research involving biopsychosocial/psychological interventions

Participants were randomised in their dyads following consent and completion of the baseline assessments. A simple randomisation procedure was provided by the East Midlands Research Design Service. They provided the allocation sequence in opaque sealed envelopes which were opened by an independent research administrator not involved in the study. A block randomisation allocation sequence was used to ensure that in blocks of ten there would be five treatment and five control participants. Participants were randomised to either:

- **Control Group: usual care** Carers randomised to the control group received the usual range of routine care and services available to them. They did not receive the biopsychosocial intervention.
- **Intervention Group: Biopsychosocial Inter-vention, plus usual care** Carers randomised to the intervention group were invited to receive the biopsychosocial intervention, in addition to usual care. It included two-hour sessions delivered once a week for six consecutive weeks. A manual was developed with specific content designed to be delivered at each of the weekly sessions.^16^ At the beginning of each session the group facilitator, who was a research psychologist, would set the scene for that particular session and participants would be given a handout from the manual which contained information about the topics to be covered. Attendees then went on to take part in practical activities which facilitated a group discussion. Topics covered in the sessions included: an introduction to stroke and caring, adjustment and mood, emotions and thoughts, dealing with problems, stress and coping, and a wellbeing action plan. A relaxation exercise was offered at the conclusion of each session which was optional for the participants to engage with. The intention was to deliver the intervention in a group format for up to five people. However, the intervention was also offered on a one-to-one basis for carers who were keen to take part in the study but were unable to attend groups; this formed part of our feasibility assessment.

The main endpoint of the study was to determine the feasibility of conducting a larger, adequately powered study. This was a composite of the feasibility objectives outlined above. The stroke carer outcomes at six months post-randomisation were: anxiety and depression; health related quality of life; and carer strain. The outcome measures used were: Hospital Anxiety and Depression Scale^19^; EuroQol EQ5D-5L^20^ and the caregiver burden scale.^21^ Stroke survivor outcomes at six months were: level of disability; ability to perform personal activities of daily living; level of anxiety and depression; health related quality of life. The outcomes measures used were: Modified Rankin Scale^22^; Barthel Index^23^; Hospital Anxiety and Depression Scale^19^ and EuroQol EQ5D-5L.^20^

Follow-up assessment visits were completed at six months by an independent research assistant who was blinded to group allocation. Other members of the research team were aware of group allocation for the purpose of running the study and delivering the intervention. Trial participants were not blinded because they needed to be informed if they were to receive the novel intervention or not.

### Qualitative interviews

In addition to the collection of feasibility data, we aimed to complete up to ten qualitative semi-structured interviews with stroke carers in each arm of the study. The purpose of the interviews was to obtain feedback on all aspects of the study. Two pre-prepared interview topic guides were developed collaboratively by the research team with the project steering group which included lay members and stroke clinicians. For those who received the intervention, the topic guide included questions on the participants’ experiences of being a stroke carer, the intervention content, group versus one-to-one delivery, and outcomes. For those in the control group, the topic guide included questions on the experiences of being a stoke carer and the nature of usual care received.

The aim was to purposively select interview participants to include carers of stroke survivors with varying degrees of severity of stroke, and carers of differing ages and genders. Interviews were conducted by the study chief investigator. The aim was to conduct these within four weeks of completion of the intervention and a corresponding timepoint for control participants. They were conducted face-to-face either in the participant’s home or on the university campus according to the participant’s preference. Interviews were audio recorded and transcribed verbatim.

### Analysis

For feasibility outcomes, descriptive statistics were calculated based on analysis of the trial screening and recruitment log, follow-up rates, and data gathered during the groups on attendance. Carer and stroke survivor outcome data was analysed by intention to treat, and participants were analysed according to their group assignment irrespective of whether they received the biopsychosocial intervention. As this was a feasibility trial, no formal sample size calculation was required. Summary statistics were used and effectiveness testing was not carried out as this is not appropriate for feasibility work.

The qualitative interview data was initially coded and analysed using the principles of thematic analysis, as described by Braun and Clarke.^24^ In the first stage, all transcripts were checked for accuracy and two researchers familiarised themselves with the data. Three transcripts were initially coded in duplicate by two researchers, who then met to compare, discuss initial codes and agree a working coding framework. The remainder of the transcripts were coded by one of the two researchers. The researchers then worked together to search for patterns in the codes to form initial themes and create the thematic analysis. Only data which is relevant to the feasibility objectives is presented here (the theme is indicated in parenthesis and italics below). Three researchers selected extracts from the wider thematic analysis to present in this paper. Extracts were selected that were relevant and pertinent to the feasibility and acceptability objectives of this paper as related to recruitment (theme: *willingness to attend intervention sessions*); timing (theme: *optimal timing of intervention*) and group versus one-to-one delivery (theme: *normalisation and group support*).

## Results

### Recruitment and participant flow

Figure 1 shows the recruitment figures and the flow of participants through the study. In total, 257 dyads of stroke survivors and their carers were approached.

Forty-one dyads of carers and stroke survivors consented and 35 were randomised, 18 to the biopsychosocial intervention and 17 to usual care control. There was a time-lag, no greater than eight weeks between consent and randomisation to allow recruitment of sufficient numbers in order to form a group. Six dyads withdrew during this period, mainly citing logistical reasons and practicalities such as “having too much on”. The characteristics of these dyads were different to those who continued with the study; the stroke survivors were all younger males with a mean (SD) age of 44 (20) years and three of the carers were wives who all had work responsibilities.

### Baseline data

The demographic details for the carers and stroke survivors are shown in Table 1. Table 2 shows the baseline measures by group allocation. Carers in the biopsychosocial intervention group had a slightly higher quality of life score at baseline, but the two arms were well matched on other measures. Stroke survivors with carers in the biopsychosocial intervention group had higher quality of life and lower anxiety and depression scores at baseline.

**Table 1.** Baseline demographic characteristics of carers and stroke survivors recruited.

	Carer		Stroke survivor	
	Intervention (*n* = 18)	Control (*n* = 17)	Intervention (*n* = 18)	Control (*n* = 17)
**Age *(SD)***	63.33 (12.72) (Range: 30–82)	61.88 (13.36) (Range: 39–80)	69.17 (12.11) (Range: 42–86)	71.47 (16.02) (Range: 27–90)
**Gender**				
Male	8 (44%)	1 (6%)	8 (44%)	12 (71%)
Female	10 (56%)	16 (94%)	10 (56%)	5 (29%)
**Marital status**				
Single	1 (6%)	1 (6%)	2 (11%)	2 (12%)
Married/partnered	17 (94%)	16 (94%)	14 (78%)	7 (41%)
Widowed/divorced/separated	0 (0%)	0 (0%)	2 (11%)	8 (47%)
**Living arrangement**				
With spouse/partner	17 (94%)	15 (88%)	14 (78%)	7 (41%)
With relatives	0 (0%)	0 (0%)	1 (6%)	1 (6%)
Alone	1 (6%)	2 (12%)	3 (16%)	7 (41%)
Other	0 (0%)	0 (0%)	0 (0%)	2 (12%)
**Employment (pre-admission)**				
Full-time	3 (17%)	5 (29%)	4 (22%)	3 (18%)
Part-time	3 (17%)	1 (6%)	0 (0%)	0 (0%)
Unemployed	0 (0%)	0 (0%)	0 (0%)	1 (6%)
Retired	10 (56%)	9 (53%)	14 (78%)	13 (76%)
Other	1 (5%)	0 (0%)		
Unpaid	1 (5%)	2 (12%)		
**Ethnicity**				
White British	17 (94%)	16 (94%)	17 (94%)	16 (94%)
Other	1 (6%)	1 (6%)	1 (6%)	1 (6%)
**Relationship to patient**				
Partner	13 (72%)	7 (41%)		
Child	3 (17%)	3 (17%)		
Parent	0 (0%)	2 (12%)		
Sibling	2 (11%)	1 (6%)		
Other relative	0 (0%)	2 (12%)		
Unrelated other	0 (0%)	2 (12%)		
**Acting as carer (pre-admission)**				
Yes			3 (17%)	3 (17%)
No			15 (83%)	14 (83%)
**Lateralisation of stroke**				
Left			14 (78%)	8 (47%)
Right			3 (16%)	8 (47%)
Not known			1 (6%)	1 (6%)
**Stroke type**				
Ischaemic			12 (67%)	13 (76%)
Haemorrhagic			6 (33%)	4 (24%)
**Side of weakness**				
Left			2 (11%)	7 (41%)
Right			15 (83%)	5 (29%)
Bilateral			0 (0%)	4 (24%)
Unknown			0 (0%)	1 (6%)
**Previous stroke(s)**				
Yes			1 (6%)	2 (12%)
No			17 (94%)	15 (88%)
**Discharge destination**				
Home			13 (72%)	16 (94%)
Early supported discharge			2 (11%)	0 (0%)
Intermediate care			3 (17%)	1 (6%)
**Time (days) from stroke to recruitment *(SD)***			84.11 (55.39)	76.83 (57.32)
**Time (days) from stroke to discharge *(SD)***			41.5 (38.27)	35.29 (41.47)

**Table 2.** Baseline measures of carers and stroke survivors recruited: Mean *(SD)*.

	Carer		Stroke survivor	
	Intervention (*n* = 18)	Control (*n* = 17)	Intervention	Control
EQ5D	0.77 (0.22)	0.73 (0.30)	(*n* = 15) 0.56 (0.29)	(*n* = 15) 0.44 (0.33)
EQ5D perceived health	74% (17.39)	75% (15.31)	(*n* = 16) 65% (18.20)	(*n* = 15) 64% (20.13)
HADS TOTAL* HADS anxiety* HADS depression*	13.17 (8.23) 9 (5.36) 4.17 (3.73)	15.11 (8.66) 9.82 (5.33) 5.29 (4.62)	(*n* = 14) 12.79 (9.43) 7.08 (6.22) 5.71 (4.45)	(*n* = 15) 19 (9.51) 8.47 (5.36) 10.53 (6.07)
Carer burden scale*	28.56 (18.2)	29.24 (18.3)		
Modified rankin score*			(*n* = 18) 3.72 (0.83)	(*n* = 15) 3.06 (1.44)
NIHSS			(*n* = 13) 9.08 (8.1)	(*n* = 11) 8.64 (9.3)
Barthel index			(*n* = 18) 11.78 (6.58)	(*n* = 16) 10.75 (7.06)
MOCA			(*n* = 11) 18.55 (7.08)	(*n* = 10) (8.80)

* higher score indicates poorer outcome.

EQ5D: EuroQOL five dimensions questionnaire; HADS: Hospital anxiety and Depression Scale; NIHSS: National Institutes of Health Stroke Scale; MOCA: Montreal Cognitive Assessment.

Interviews were completed with six intervention participants and five control participants. We were unable to fulfil our target of ten interviewees per arm due to the demands on the carers time. Demographic details of interviewees are shown in Table 3. There were more male carers interviewed in the intervention arm and two participants chose to be interviewed together. Of those interview participants who had received the intervention, two had received the intervention on a one-to-one basis, and the rest had received the intervention in the group format.

**Table 3.** Qualitative interview participant demographics.

	Intervention (*n* = 6)	Control (*n* = 5)
**Gender**		
Male	4	1
Female	2	4
**Age (mean)**	70.1 (11.7)	68.8 (9.5)
**Employment**		
Retired	4	4
Full-time	2	1
**Relationship to stroke survivor**		
Spouse	5	2
Daughter	1	1
Son		1
Sister-in-Law		1
**Mean time (months) in carer role at time of interview *(SD)***	7.8 (1.8)	10.4 (6.1)
**Previous experience in the carer role**		
Yes	2	2
No	4	3

### Feasibility outcomes

We experienced difficulties in identifying eligible dyads who were willing to enter the study. The trial was open to recruitment for 19 months during which time 41 dyads consented. This was 16% of those approached, and an average of two per month. During the initial recruitment stage from November 2015 to August 2016 only six dyads were recruited, five of whom subsequently withdrew. Key barriers identified by clinical staff in the early stages of recruitment were finding eligible dyads where the stroke survivor had the mental capacity to consent and carers who were potentially interested in participating, as well as feeling the approach was too early.

An ethical amendment was obtained to recruit stroke survivors without capacity via consultee opinion, as this had not been part of our original protocol. At the start of the study we were identifying potential future participants from stroke units only but later expanded the recruitment strategy to approach dyads at the six-week follow-up in outpatient clinics and from the community stroke services. In total, 27 of the 41 dyads (66%) were identified at six-week outpatient clinics reflecting the success of this change in recruitment strategy.

Findings from the qualitative data suggested that the degree of stroke severity had an influence on carers’ willingness to attend intervention sessions and therefore take part in the study. They needed to feel able to leave the stroke survivor:

*“She’d reached a stage where she could stay for a couple of hours on her own and I did ring. . . I would be able to leave her now for two or three hours. Not. . . worrying too much about her.”* (Carer 5, Husband)

Contrary to the recruitment challenges in identifying potential participants early on the stroke unit, the qualitative data suggested that in relation to optimal timing of intervention the sessions would have been helpful had they been received earlier, particularly the stroke education elements. This is also consistent with recruitment data from the six-week follow-up clinics as ten carers did not consent to take part, reporting that they had needed the intervention earlier (Figure 1).

*“I think the nearer perhaps to the event, the better really. Especially when you go through. . . what is a stroke.”* (Carer 1, Wife)

Table 4 shows the baseline characterises of those allocated to the intervention group and the number of sessions attended.

**Table 4.** Group & one-to-one delivery characteristics and session attendance.

	Group delivery (*n* = 14)					One-to-one delivery (*n* = 4)
		Group 1	Group 2	Group 3	**Total**	
Mean age *(SD)*		64 (14)	61 (15)	58 (14)	**61 (3)**	71 (8)
Gender (F/M)		3/2	3/1	4/1	**10/4**	1/3
Mean number of sessions attended *(SD)*		3.2 (2.58)	3.25 (2.52)	3.6 (1.29)	**3.36** **(1.98)**	5.75 (0.5)
Number Allocated		5	4	5	**14**	4
Relationship to survivor (%)	Spouse	3	3	3	**9**	4
	Sibling	0	1	1	**2**	0
	Child	2	0	1	**3**	0

The bold face represents the total scores for the group delivery metrics.

**Figure 2.**
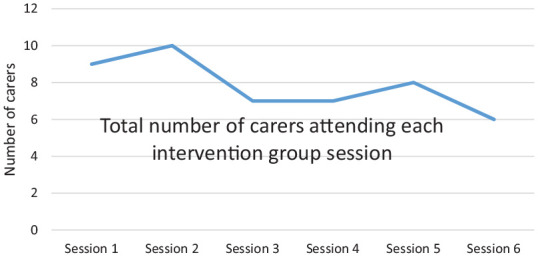
Attendance of carers at intervention group sessions. 14 carers received group intervention (4 had 1:1 intervention). Y axis shows number of carers/14 attending session 1, 2, 3 etc (X axis).

Figure 2 shows the attendance at groups by session number. The primary reason for non-attendance was that they were unable to balance the intervention sessions with other commitments, which included: caring for the stroke survivor, childcare and work.

#### Acceptability of the intervention

Notwithstanding an average attendance at the groups of three sessions, participants reported that they valued the intervention overall. Intervention participants described the emotional validation afforded through the sharing of experiences between group members and receiving peer support. This normalisation and group support was an important component of the intervention:

*“. . . listening to each other’s experiences being a carer. . . you think well ‘we’re not on our own in this’, you know there’s other people gone through it more or less that identical situation.”* (Carer 4, Husband)

One-to-one sessions were much better attended, likely due to greater flexibility of time and place of the session though one participant who received the intervention one-to-one would have preferred to receive the intervention within the group:

*“. . .there are lots of advantages to meeting on a one-to-one but they are just purely organisational advantages. . .you get a lot more support from others in a group setting.”* (Carer 6, Husband)

Aspects reported to improve the acceptability of the intervention and its delivery (in carer interviews) included: delivery after routine stroke services have ended; an accessible venue with good public transport links; delivery outside the hospital environment. Overall acceptability was high; however, some carers felt that the programme was too long and suggested the number of sessions could have been reduced. Furthermore, the aspect of the intervention that was highlighted as most lacking was a focus on practical support and signposting; participants reported struggling to know where to go to get help with benefits, equipment, and transport.

#### Suitability of outcome measures

There were improve-ments from baseline in both groups for stroke carers and stroke survivors on all outcome measures, as shown in Table 5. In both groups, the improvements from baseline were relatively large; this likely reflects the acute stage at which dyads were recruited and their subsequent recovery. Overall, there was a slightly greater improvement in the control group.

**Table 5.** Six-month follow-up measures of carers and stroke survivors recruited: Mean *(SD)*.

	Carer		Stroke survivor	
	Intervention (*n* = 17)	Control (*n* = 13)	Intervention	Control
EQ5D	0.88 (0.14)	0.91 (0.10)	(*n* = 13) 0.65 (0.32)	(*n* = 11) 0.73 (0.2)
EQ5D Perceived Health	76% (15.36)	83% (8.96)	(*n* = 13) 65% (22.86)	(*n* = 11) 66% (20.83)
HADS TOTAL* HADS anxiety* HADS depression*	8.94 (4.66) 5.71 (3.26) 3.24 (2.84)	8.15 (4.38) 4.85 (2.58) 3.31 (2.90)	(*n* = 13) 10.85 (6.79) 6.92 (5.31) 3.92 (2.60)	(*n* = 10) 9.5 (6.2) 4.3 (2.98) 5.2 (3.77)
Carer burden scale*	18.71 (14.57)	16.77 (12.03)		
Modified rankin score*			(*n* = 14) 2.07 (1.59)	(*n* = 12) 2.33 (1.44)
Barthel index			(*n* = 14) 15.57 (5.52)	(*n* = 12) 15.83 (6.58)

* higher score indicates poorer outcome.

EQ5D: EuroQOL five dimensions questionnaire; HADS: Hospital anxiety and Depression Scale; NIHSS: National Institutes of Health Stroke Scale; MOCA: Montreal Cognitive Assessment.

The outcome measures focussed on health related quality of life, anxiety and depression, and carer strain. However, carers reported that it was the opportunity to think about problems in different ways that influenced their ability to cope. This appeared to be the primary way the intervention affected them:

*“. . .it’s a matter of being able to cope with it. So long as you can cope with it, you can sort of laugh it off as you might say.”* (Carer 4, Husband)

## Discussion

This was a feasibility trial of an intervention to support the wellbeing of carers of stroke survivors. We exceeded our recruitment target of 40 dyads; delivered the intervention to stroke carers in both a group and one-to-one format; and followed-up 86% (30) of stroke carers. The intervention was largely acceptable to participants who particularly valued the peer support in the group format. Although we demonstrated that such a trial is feasible in terms of recruitment and follow-up, we have identified a number of issues in relation to research involving carers as part of a randomised controlled trial.

We experienced difficulties identifying eligible participants who were willing to be randomised, and this was particularly evident whilst the stroke survivor was still an inpatient. It was also difficult for people to identify themselves as ‘carers’ before their role in the home had been established post-discharge. Furthermore, a number of carers did not agree to take part in the study as they reported that they had insufficient time to participate in the intervention. This was also observed after recruitment as some participants who were randomised to receive the intervention missed sessions due to balancing caring commitments and other appointments. The timing of approach and mode of delivery of a carer intervention appears critical. Recruitment early after stroke proved challenging and yet in the post-intervention qualitative interviews several participants reported that they would have valued the intervention earlier. This finding is also consistent with findings from focus groups we conducted with stroke carers prior to the study commencing,^25^ who felt the intervention should be offered early in the stroke pathway.

The content of the intervention was largely well received although interviews suggested that a shorter programme would have been better. It is possible that certain components of the intervention could be delivered on-line allowing the individual to access this information as and when required. Peer support also appeared to be a core success component of the programme with participants telling us they particularly valued the interaction with other carers and that this normalised how they were feeling. Given competing interests on carers time this interaction may also be possible to deliver as an on-line group using technologies such as skype or zoom. However it is acknowledged that access to such technology and confidence in its usage may be potential barriers for this mode of delivery. Care arrangements for stroke survivors provided nearby the group venue and a rolling programme of the biopsychological intervention may also offer possible solutions enhancing attendance at all sessions.

The principal strength of this study is that it was an empirical study that developed and tested the feasibility of a biopsychosocial intervention specifically for carers of stroke survivors. The intervention was developed based on available evidence from the literature whilst incorporating the opinions and expertise of stroke carers and stroke clinical and research experts.^16,25^ However providing alternative methods of delivery (groups vs one-to-one) could be extremely challenging if embarking on a large scale trial and would need to be clearly thought through taking randomisation procedures, power calculations, workforce requirements and costs into consideration.

A key limitation of the study is that the trial was conducted in a single site and further issues may have arisen if conducted elsewhere. The greatest challenge we faced was recruitment of participants however our difficulties are not unique and indeed our recruitment of two participants per month is in line with a recent systematic review.^26^ Similarly, the attendance levels seen within the group intervention are reflective of attendance levels for other face-to-face group therapy interventions.^25,27^ Although initial contact on the stroke unit proved challenging and resulted in difficulties in recruitment, conversely a delayed approach in some cases resulted in the intervention being delivered after the time that it was reported as most needed. Timing of approach for the delivery of the intervention would ideally be bespoke, particular to the individual’s situation and readiness for engagement.

The most suitable primary outcome measure remains unclear. As the intervention was delivered relatively early in the stroke pathway, it was intended to be a supportive intervention with the aim of keeping more significant issues such as depression, at bay. Whilst prevention of mental health problems is of paramount importance, the primary effect appeared to be the ability to cope and/or enhanced wellbeing. Therefore, it may be judicious to focus on a measure of coping ability in any future trial rather than health related quality of life or anxiety and depression.

Our results suggest that bespoke timing and tailoring may enhance successful and appropriate delivery of such an intervention. Further work to test the delivery of this biopsychosocial intervention is needed. There remains a requirement to ensure that the needs of carers are adequately addressed in order to prevent deterioration in their own health and wellbeing and enable them to continue to provide support to stroke survivors in the community.

**Clinical messages**

- It is feasible to deliver an intervention informed by biopsychosocial principals designed to support a stroke carer in both a group and one-to-one format.
- Timing of approach and mode of delivery is critical.
- Peer support in normalising feelings encountered in caring for a stroke survivor is particularly valued.
